# Edema by T2-weighted imaging in salvaged myocardium is extracellular, not intracellular

**DOI:** 10.1186/1532-429X-13-S1-P70

**Published:** 2011-02-02

**Authors:** Martin Ugander, Paul S Bagi, O Julian Booker, Li-Yueh Hsu, Abiola J Oki, Andreas Greiser, Peter Kellman, Anthony H Aletras, Andrew E Arai

**Affiliations:** 1 National Institutes of Health, Bethesda, MD, USA; 2 Siemens AG Healthcare Sector, Erlangen, Germany

## Introduction

T2-weighted MRI can image myocardial edema in salvaged, non-infarcted myocardium. However, it is unclear whether this edema is mostly intra-, extracellular, or balanced.

## Purpose

We tested the hypothesis that edematous non-infarcted myocardium would show a balanced increase in both the intra- and extracellular volume.

## Methods

Dogs (n=10) underwent coronary occlusion (2h) and reperfusion (4h). 1.5T CMR images (Siemens) were taken before and 45 minutes into contrast administration (0.15mmol/kg Gd-DTPA bolus followed by 0.003mmol/kg/min infusion). Myocardial T1 was measured by modified Look-Locker imaging (MOLLI), infarction by late gadolinium enhancement (LGE) imaging, and edema by T2-prepared SSFP. T1 and R1 (1/T1) pixel maps were generated from MOLLI images. Contrast agent concentration is proportional to change in R1 between pre- and post contrast. ECV was defined as [1-hematocrit] * [DeltaR1myocardium (R1post-R1pre contrast)]/[DeltaR1blood], thus yielding a quantitative pixel map of the ECV ranging from 0-100%. Change in left ventricular mass between baseline and post-reperfusion was quantified by cine SSFP imaging. The change in non-contrast tissue T1 by T1-mapping was assumed to linearly reflect swelling for both the region of infarction determined by LGE and salvaged myocardium at risk determined by non-contrast T1 mapping. In combination with ECV measurements, these measurements allowed for quantification of the absolute myocardial intra- and extracellular volume at baseline and in salvaged myocardium. Data is presented as mean±SD.

## Results

**Figure 1:**
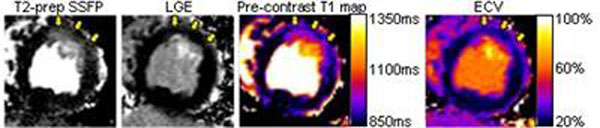
Edema in non-infarcted, salvaged myocardium (arrows) displays an increased extracellular volume by quantitative ECV imaging.

Following reperfusion, edema in salvaged myocardium showed an increase in extracellular volume (4.0±1.7 vs. 2.4±1.1 ml, p=0.006, n=9) but no increase in intracellular volume (7.5±3.3 ml vs. 7.5±3.3 ml, p=1.0, n=9) compared to pre-occlusion baseline. Consequently, salvaged myocardium (Arrows in Figure 1) had an ECV of 34±7%, which was greater than normal myocardium (24±3%, p=0.04, n=10) and less than infarcted myocardium (58±13%, p<0.001, n=10). T1 of edematous salvaged myocardium was greater than normal myocardium before contrast administration (1050±114ms vs 919±66ms, p<0.001, n=10) and lesser after contrast (461±57ms vs 512±67ms, p<0.001, n=10).

## Conclusions

Edema in salvaged non-infarcted myocardium is extracellular, not intracellular. The edema is not balanced. This edema may be difficult to differentiate from normal myocardium by LGE imaging because of relatively small differences in post-contrast T1. Since baseline T1 is different, edematous salvaged myocardium enhances more than normal myocardium and more than predicted by only post-contrast LGE images. This explains why salvaged non-infarcted myocardium is not apparent on LGE images.

